# What Predicts Stable Mental Health in the 18–29 Age Group Compared to Older Age Groups? Results from the Stockholm Public Health Cohort 2002–2014

**DOI:** 10.3390/ijerph15122859

**Published:** 2018-12-14

**Authors:** Regina Winzer, Kimmo Sorjonen, Lene Lindberg

**Affiliations:** 1 Department of Public Health Sciences, Karolinska Institutet, 17165 Stockholm, Sweden; lene.lindberg@ki.se; 2 Department of Living Conditions and Lifestyles, Public Health Agency of Sweden, 17182 Solna, Sweden; 3 Department of Clinical Neuroscience, Karolinska Institutet, 17165 Stockholm, Sweden; kimmo.sorjonen@ki.se; 4 Center for Epidemiology and Community Medicine, Stockholm County Council, 10431 Stockholm, Sweden

**Keywords:** longitudinal study, young adults, stable mental health, social determinants

## Abstract

Mental health has decreased in young people since the 1990s, and mental health promotion is an urgent matter. A first step is to identify which social determinants could be of importance for intervention. We used the Stockholm Public Health Cohort, a longitudinal population-based health survey, completed by 31,000 inhabitants in the Stockholm County. We focused on the 18–29 age group, *n* = 3373 (60% females, 40% males) and aimed at assessing which social determinants predict stable mental health, measured as scoring <3 points on the General Health Questionnaire 12 at all time points: 2002, 2007, 2010, and 2014. Forty-six percent of males and 36% of females reported stable mental health. Among the 17 predictors on sociodemographics, socioeconomics, social capital, health behavior, and victimization, six predicted stable mental health in the following order: occupation and especially employment, emotional support, male gender, being born in Sweden, absence of financial strain, and consumption of fruit and berries. In the 30–84 age group, 66% males and 55% females reported stable mental health. Nine determinants in the following rank predicted stable mental health: absence of financial strain, occupation and especially being self-employed, emotional support, male gender, physical activity, instrumental support, interpersonal trust, community trust, and absence of hazardous alcohol consumption. Interaction analysis showed significant difference between the younger and older group regarding physical activity and absence of financial strain with importance being higher for the older group. Our findings indicate that the determinants of health differ across the life-course with fewer predictors related to social capital and health behavior in the younger group compared to the older. We conclude that health-promoting interventions should be lifespan-sensitive.

## 1. Introduction

Promoting mental health and preventing mental ill-health in populations has been a public-health issue for decades. Young people have been especially in focus since a decrease in their mental health in many Western societies has been observed since the 1990s [1,2,3,4,5], and internalizing problems in adolescents, especially in girls, seem to have increased during the 21th century [6]. For self-rated mental ill-health in adolescents, a global rise is evident between 1980 and 2000, with stabilization between 2000 and 2010. However, an exception is observed for Northern Europe, where a continuous increase in adolescents’ mental and somatic complaints in 1982–2013 has been shown [7]. Compared to older age groups, young adults 16–29 years in Sweden report the highest levels of psychological distress, 54% in women and 33% in men, according to the National Public Health Survey 2016 [8]. This pattern is similar to other Nordic countries as Denmark [9] and Finland [10].

Less well-known, and in contrast to studies about social determinants of health [11], is the fact that symptoms of mental ill-health also highly affect students during their tertiary education. According to systematic reviews, the prevalence of depressive symptoms among students varies between 27.2% and 30.6% [12,13], with even higher rates for anxiety and distress [14,15]. Furthermore, students in higher education report up to 4.4 times higher distress than age-matched peers do [14,16], significantly higher rates compared to those actively working [17], and elevated levels compared to the general population [12,13].

To develop positive mental health during childhood, adolescence, and young adulthood is an important outcome in its own right, but it also constitutes a predictor for educational attainment and perceived competence [18], future income levels [19], personal finances [20], occupational attainment, life satisfaction, and quality of relationships [21] in later life.

When applying the biopsychosocial model [22], it becomes evident that it is possible to promote mental health by an array of actions, e.g., through the social determinants of health. Social determinants have considerable impact in peoples’ lives [23,24] and are important predictors for general health [25] as well as for mental health [26]. A review by Allen et al. on the determinants for mental health indicates a social gradient, meaning that worldwide mental distress affects those with lower socioeconomic status proportionally worse compared with those with higher status. Furthermore, female gender and low educational attainment, low household income, financial debt, and unemployment also have an association with mental distress and disorder [11]. However, the social determinants of health also include features related to the concept of social capital, e.g., participation in organizations, emotional and social support, trust, and social harmony [27]. A large body of research indicates that health behavior factors—i.e., physical activity [28,29], tobacco and alcohol consumption [30,31], and nutrition [32,33,34]—are also associated with mental health. A problem in the study of social determinants of health is that those factors available to analyze account for risk factors for mental ill-health rather than promotive factors for mental health. Though they may be related, risk and promotive factors have diverse features, i.e., they do not represent different sides of the same coin [35,36].

Regarding factors related to social capital, previous studies have found associations with young people’s positive development and mental health. Hawkins et al. found that social-capital constructs, such as civic action and engagement, trust and tolerance of others, and trust in authorities and organizations, were consistently related to positive development in 19–20-year-old young adults [37]. In the same cohort, precursors for positive development in emerging adulthood were investigated and identified as the following: stronger family and peer relationships, better adjustment to school settings, higher family socioeconomic status, and better emotional control [38]. Findings on the association between immigrant status and mental distress are inconclusive. Some studies indicate that immigrants perceive less distress compared to nonimmigrants [39,40], while a systematic review found higher levels of mental ill-health in immigrants in 13 of 21 included studies. The authors concluded that the differences in mental distress versus native populations could be related to stressful conditions during the migration process and in the host countries [41]. A longitudinal study found that ethnic minority status in adolescents, despite economic disadvantage and racism, predicted a better mental health outcome at 21–23 years of age compared to the British majority. A mediating factor for this association was family connectedness and support [42]. In contrast, poor family relationships in adolescence predicted mental ill-health later in life [43]. Social support together with internal resources were also positively correlated with mental health in a cross-sectional study among university students, while perceived stress showed a negative association with mental health [44]. According to an overview article, chronic arousal due to stress has been identified as a key factor for mental ill-health irrespective of age group [26]. Victimization in young people differs between males and females regarding their perpetrators and places of victimization, but their adverse mental-health associations are similar [45,46,47] and have longstanding consequences for adulthood [48].

As shown, many studies have focused on mental ill-health, whereas mental health has been examined to a lesser extent. However, with the exception of one study, which investigated the absence of a diagnosis as a measure for endurance in mental health [21], according to our knowledge, stability in mental health has not been investigated as an effect of former health determinants, though stability in mental health seems to be an important factor for future positive and negative life events. In a longitudinal study, Keyes et al. found that relatively small declines in mental health predicted future mental illness, and the researchers underscored the importance of investing in mental-health promotion and protection [49]. Another longitudinal study revealed that seven-year mortality was more strongly predicted by the absence of positive wellbeing than the presence of psychological symptoms [35]. With our study, we wanted to examine sociodemographic factors, social capital, and health behavior and exposure to violence, and their association with the stability of mental health.

The aim of this study was to assess which determinants of health contribute to a stable mental health in the 18–29 age group. More precisely, we wanted to investigate the determinants contributing to a state free from psychological distress. Furthermore, we investigated if these determinants differ from those observed in the older population, 30 years and older.

## 2. Methods

### 2.1. Sampled Participants and Procedures

The sample consisted of the Stockholm Public Health Cohort (SPHC), a population-based postal and/or web survey set up in 2002. The cohort profile, including the questionnaire, has been described in detail elsewhere [50]. In brief, the survey included an area-based random sample of 50,000 inhabitants in the Stockholm County aged 18–84 years in 2002, and was followed up in 2007, 2010, and 2014. The response rate was 62% in 2002 (*n* = 31,182), and the resurveyed rates for the follow-up years were 79, 77, and 71%, respectively. Data collection was conducted by Statistics Sweden in collaboration with the Department of Public Health Sciences at Karolinska Institutet. Survey data were supplemented by register data, and participants gave their informed consent to future contacts and record linkages. The study was approved by the Stockholm Regional Ethical Review Board (no. 2010/1879-31/5, no. 2007/545-31, no. 2018/1569-32).

**Figure 1:**
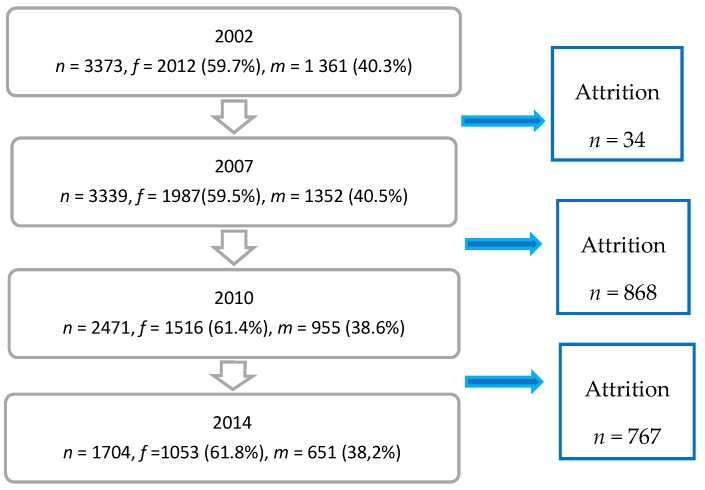
Flow-chart of respondents in the 18–29 age group of the Stockholm Public Health Cohort 2002, 2007, 2010, and 2014.

We used data for the 12-year period, 2002–2014, and analyzed the 18–29 age group. In 2002, our age group comprised 3373 persons, 2012 women (59.7%) and 1361 men (40.3%), equaling a response rate of 53.9%. For the following surveys, see Figure 1. As a non-response analysis was not available we were able to comment on the attrition characteristics.

The 18–29 age group was compared with the rest of the population, 30 years and older, 7237 men (43.6%) and 9377 women (56.4%). (Supplementary Materials, Figure S1. Flow-chart of the respondents in the 30 years and older age group of the Stockholm Public Health Cohort 2002, 2007, 2010, and 2014).

In the following, we present our data using the STROBE Statement and provide the checklist of items that should be included in reports of cohort studies (Supplementary Materials, Table S1).

### 2.2. Study Variables

Our outcome variable, absence of psychological distress, was measured by the General Health Questionnaire (GHQ-12). This 12-item self-report questionnaire, which originated from earlier versions (60, 30, and 28 items), was created to assess psychiatric morbidity in clinical and community settings [51]. The instrument has been validated and applied in various international population surveys [52,53,54,55,56], as well as in Swedish contexts [57,58]. The included items of the instrument capture symptoms of anxiety, depression, social dysfunction, and stress-related problems, i.e., psychological distress. They have a severity range from 0 = better than usual, to 3 = much worse than usual. Following the results of a recent validation [58], we used the Standard GHQ-12 Scoring method, in the range of 0–12 points. Following earlier analyses of the same cohort, a cut-off of ≥3 points was chosen for symptoms of mental ill-health [59]. We defined ‘absence of psychological distress’ as a GHQ-12 score < 3 at all four measuring points, 2002, 2007, 2010, and 2014 referred as ‘stable mental health’ in the following.

A comparison was made with those reporting psychological distress at one to three time points during the four measuring points, referred as ‘unstable mental health’. However, we excluded the respondents reporting psychological distress at all four measurement points (i.e., a constant stable negative mental health) as we considered the numbers being too small to be included in the statistical analyses. The stable mental health and unstable mental health groups were compared separately in the 18–29 and 30–84 age groups in order to assess if the determinants among the younger and older age groups differed from each other. The potential social determinants of health—i.e., the survey items, their response alternatives, and categorization measured in 2002—are described and commented in Supplementary Materials, Table S2.

### 2.3. Statistical Analysis

Logistic regression analysis predicting stable mental health was conducted separately for the 18–29 age group and the 30 years and older age group (Table 1). For missing data no imputations were carried out. Case-wise deletion, the default in SPSS, was employed, i.e., only respondents with complete data were included in the analyses. For 15 out the 17 variables, the attrition rate varied from 0.0–2.8%. Attrition was highest for community trust, with 7.5% in the younger group, and 8.4% in the older group, and further, hazardous alcohol consumption, with 9.7% and 10.2%, respectively.

Interaction analysis was performed for presumptive differences in health determinants between the two groups (Table 1). All analyses were performed on IBM/SPSS Statistics version 22.0 (IBM Corp., Armonk, NY, USA). We used the level of statistical significance at *p* < 0.05.

## 3. Results

Out of the 18–29-year-old study population, i.e., 3373 individuals (Figure 1), the GHQ-12 was completed by 2471 individuals. According to frequency analyses for the 18–29 age group, stable mental health was reported by 434, i.e., 46.2% out of a total of 939 men, and 524, i.e., 35.7% out of 1466 women. Sixty-six individuals reporting stable mental ill health were excluded from the analysis. In the 30–84 age group, i.e., 20,421 individuals (Supplementary Materials, Figure S1), the GHQ-12 was completed by 16,857 individuals. In our corresponding frequency analyses, stable mental health was reported by 4757, i.e., 65.7% out of a total of 7237 men, and 5140, i.e., 54.9% out of 9377 women for the 30–84 age group. In total, 243 individuals reporting stable mental ill health were excluded from the analysis. All 17 potential determinants were included in the regression analysis presented in Table 1.

The analysis showed that occupational status was the strongest predictor for stable mental health for 18–29 years. Different kinds of occupational status showed significant difference in odds for correlation with stable mental health compared to unemployment. The most robust associations were found for employment (OR = 2.63, 95% CI = 1.46–4.76), leave of absence/parental leave (OR = 2.54, 95% CI = 1.15–5.63), and being a student (OR = 1.93, 95% CI = 1.06–3.53). High emotional support, compared to low emotional support, appeared to be the second strongest predictor for stable mental health (OR = 1.95, 95% CI = 1.08–3.53). Furthermore, the male gender was a predictor for stable mental health compared to the female gender (OR = 1.91, 95% CI = 1.54–2.36), followed by being born in Sweden compared to born outside Europe (OR = 1.86, 95% CI = 1.07–3.26). Other determinants for stable mental health were experiencing no financial strain compared to the experience of major financial strain (OR = 1.56, 95% CI = 1.07–2.28), and, lastly, daily consumption of fruit and berries compared to seldom consumption of fruit and berries (OR = 1.39, 95% CI = 1.05–1.84). Physical activity, no daily tobacco smoking, and absence of hazardous alcohol consumption were not associated with stability in mental health.

In the regressed data, the findings in the older age group contrasted from the younger, revealing that experiencing no financial strain, compared to the experience of major financial strain was a principal predictor for stable mental health (OR = 2.79, 95% CI = 2.25–3.47), as shown in Table 1. Having some, rather than major financial strain, also yielded stable mental health in the older age group (OR = 1.66, 95% CI = 1.32–2.10). Secondly, occupational status predicted stable mental health, especially the status of running one’s own business (OR = 2.25, 95% CI = 1.67–3.04), a position outside the labor market (OR = 2.21, 95% CI = 1.66–2.93), or being employed (OR = 1.70, 95% CI = 1.29–2.34) compared to unemployment. In third place, high and also moderate emotional support related to stable mental health (OR = 1.91, 95% CI = 1.62–2.27; and OR = 1.49, 95% CI = 1.27–1.74, respectively). The male gender, compared to female, ranked in fourth place (OR = 1.64, 95% CI = 1.51–1.78). Regular (OR = 1.54, 95% CI = 1.31–1.80) and moderate physical activity (OR = 1.34, 95% CI = 1.19–1.51) compared to sedentary physical behavior contributed to stable mental health. Further, high instrumental support (OR = 1.45, 95% CI = 1.19–1.78) and moderate instrumental support (OR = 1.29, 95% CI = 1.06–1.57) compared to weak instrumental support, as well as trust in terms of strong interpersonal trust (OR = 1.23, 95% CI = 1.04–1.44) compared to weak interpersonal trust, and strong community trust (OR = 1.11, 95% CI = 1.02–1.21) compared to weak community trust, were associated to stable mental health. Lastly, the absence of hazardous alcohol consumption (OR = 1.11, 95% CI = 1.02–1.21) produced stable mental health.

The interaction analyses displayed in Table 1 showed that significant differences between the younger and older groups were obvious only for three determinants of health. These were physical activity, where regular physical activity (OR = 0.65, 95% CI = 0.44–0.94) and moderate physical activity (OR = 0.65, 95% CI = 0.47–0.90) yielded a difference and were of importance for a stable mental health only in the older age group. The effect of absence of financial strain (OR = 0.53, 95% CI = 0.35–0.80) also differed between the younger and older groups, and was more important among the latter. Although to refrain from tobacco smoking was not a significant predictor for stable mental health in either age group, the effect differed significantly between the groups (OR = 0.70, 95% CI = 0.50–0.97), with smoking being a more important predictor in the younger group (OR = 0.72, 95% CI = 0.51–1.01).

## 4. Discussion

Our results show that every second male and every third female in the 18–29 age group reported stable mental health in the period of 2002–2014. Our findings may be compared with the Dunedin Cohort, where only 17% of the participants (57% males) at no time point did fulfill any criteria for a psychiatric diagnosis during the six waves of investigation, i.e., a period of nearly 30 years [21]. Six determinants were associated with this stability, namely, occupational status and especially employment, being absent or on parental leave, emotional support, the male gender, being of Swedish origin, experiencing no financial strain, and having healthy food intake, illustrated by a regular consumption of fruit and berries. It is noteworthy that only one of the determinants could be assigned to social capital and health behavior, emotional support and nutrition, respectively, whereas physical activity, the absence of daily tobacco smoking and of hazardous alcohol consumption, were not of importance for stability in mental health. These findings are comparable with those in an Australian cohort study, where positive development in young adulthood was to a very small extent associated with trust in authorities, civic action, and engagement in social groups [60]. Another study of the same cohort showed, in accordance with our study, that hazardous alcohol use had no relationship with positive development in young people [61]. The authors suggest that alcohol use among young people is rather normative and does not compromise positive functioning in other domains, an explanation shared by our findings. Regarding our results on the association between the regular consumption of fruit and berries and a stable mental health may be seen as a proxy for a healthy nutritional behavior on the whole. There is emerging evidence on healthy nutrition indicated by the consumption of fruit and vegetables as a factor associated with positive mental health and wellbeing [62,63,64]. Emerson and Carbert showed in their large sample of immigrants in Canada that a higher intake of vegetables and fruit was associated with lower odds of having psychiatric disorders, lower levels of distress, and higher levels of self-rated good mental health. Furthermore, these protective findings for nutrition and mental health were independent of sociodemographic, physical health, and health behavior such as physical activity and alcohol consumption [65].

Not surprisingly, occupational status in terms of employment is the main factor predicting future stability in mental health and unemployment may cause mental ill-health according to meta-analyses [66]. Employment entails a steady income and enables a young person to establish themselves in society. According to cross-sectional studies, employment has been associated with better mental health compared to being a student [14,16,17,67].

In line with many studies and a systematic review, emotional support is of profound importance for mental health and associated with protection from depression during one’s lifespan [68,69]. Furthermore, in accordance with our findings, emotional support, but not practical support, had a stress-buffering effect in a longitudinal sample of young people [70]. The inequity in self-reported mental health between males and females, in favor of males, has been reported in research worldwide [71,72] and is also confirmed by us. Absence of financial strain also yielded stability in mental health, which could be seen as self-evident. However, the association between stability in mental health and healthy nutrition, illustrated by the consumption of fruit and berries, may not be well-known, though there is emerging evidence on this correlation [64]. Comparing our results of the younger group with the 30 years and older age group, stable mental health was experienced by a higher proportion in both males and females, which is in line with earlier findings, indicating less psychological distress in older age groups compared to the younger [73,74]. In contrast to the younger age group, more determinants related to social capital—beyond emotional support, instrumental support, interpersonal trust, and community trust—were also of importance for the older group. Furthermore, absence of financial strain was a more important determinant in the older age group compared to the younger, while country of birth had no significant association with stable mental health. Running one’s own business—i.e., being self-employed—seems to be of pronounced importance for the older group and their stability in mental health. Gevaert et al. investigated the phenomenon of self-employed people’s wellbeing in 28 European countries [75]. Their findings highlight individual characteristics, such as motivation and the ability to identify opportunities, and, furthermore, that medium and large employers present high levels of wellbeing, but not self-employed in dependencies, such as farmers and freelancers. The fact that a position outside the labor market favored a stable mental health in the older group may be surprising. However, the 30 years and older age group, on the whole consisted of 26% outside the labor market; among those above 65 years of age, 91% were in that position, most likely pensioners. Health behavior, especially physical activity and absence of hazardous alcohol consumption, seems essential for stability in mental health at older age. Physical activity was critical for the older group and showed, together with financial strain, a significant difference between the younger and older groups. We hypothesize that a reason for the significance of physical activity in the older age group might be, that sedentary behavior often increases with age and has to be compensated by a choice for physical activity in order to achieve stable mental health. Regarding the importance of absence of financial strain in the older group, we refer to Wilkinson and Pickett, illustrating how people in equivalent groups compare one selves with each other and how relative inequalities get “under the skin” and are sentenced as stigma [76]. In younger ages financial difficulties are common and normalized among students and those not being established in working life. However, financial strain becomes more seldom with increasing age and therefore, might be sentenced as a shame for those experiencing the status.

It may be surprising that participation in social organizations and in elections produced no association with stable mental health in either group. Fiorillo et al. in a longitudinal study on participation in social groups, demonstrated that it was not sufficient to only be active in associations or a member in organizations to present good mental health [77]. Instead, both membership and active participation were required. As our survey did not include items concerning membership, we were not able to assess to what degree our responders were active participants.

The strengths of our study include the longitudinal study design upon which the results are based. Furthermore, the population sample entails a relatively large sample size, and the items measuring the determinants of health are well-established and cover different aspects of public health, e.g., sociodemographics, socioeconomics, social capital, and health behavior. Their frequent use of population surveys enables our results to be compared with other studies. Our study also has limitations. Firstly, this is a correlational study that does not give conclusive indications of causality. For example, the found association between unemployment and unstable mental health could be due to unemployment affecting mental health, mental health affecting the likelihood of unemployment, or both unemployment and mental health being affected by some confounding factor. It would, of course, be interesting to study if actively helping people out of unemployment affects their mental health. Secondly, the determinants of health are restricted to the items available from 2002. Important data related to socioeconomics—i.e., income and education—were also not relevant to use for our young study population, as these records do not tend to stabilize until later in life. Thirdly, our outcome variable ‘stable mental health’ is not optimal, as it just expresses freedom from mental distress, but not flourishing mental health. The lack of items on positive mental health restricted us from measuring wellbeing and flourishing in this population sample, and choosing ‘stability in mental health’ was our second-best choice. Lastly, our comparison age group, 30 years and older, may be too wide. We could have divided our comparison group into three or more subgroups, but to make assessments of determinants of health and stable mental health in all specific age groups was not the aim of our study. Our focus was the 18–29 age group, and future studies should make more specific comparisons among different age groups.

## 5. Conclusions

Our findings showed that, for young people, occupational status, especially being employed, is of major importance to developing stable mental health. Other factors are emotional support, male gender, being of Swedish origin, absence of financial strain, and nutrition. These determinants differ from the older age group, where absence of financial strain is the key determinant for stability in mental health. Furthermore, occupational status, particularly running one’s own business and determinants related to social capital and physical activity were crucial for stability in mental health in the older age group. Our findings indicate that predictors for mental health differ over the life-course, and in order to address health-promoting interventions as efficiently as possible, policy-makers should be aware of which determinants to target in diverse age groups. Nevertheless, our study warrants replications.

## Figures and Tables

**Table 1:** Determinants for stable mental health in the 18–29 years and 30 years and older age groups.

Determinants	18–29 Years			≥30 Years			Interaction between the Determinant and the 18–29 Year and ≥30 Year Age Groups		
	OR	95% CI	Sig.	OR	95% CI	Sig.	OR	95% CI	Sig.
Gender									
Male (Reference: female)	1.91	1.54–2.36	0.000 ***	1.64	1.51–1.78	0.000 ***	1.00	0.81–1.24	0.975
Country of birth			0.111			0.474			0.266
Sweden	1.86	1.07–3.26	0.029 *	1.02	0.80–1.29	0.901	1.60	0.88–2.88	0.121
Other Nordic Countries	1.46	0.52–4.09	0.476	1.15	0.87–1.51	0.330	1.07	0.37–3.07	0.898
Other Europe	1.26	0.54–2.95	0.597	1.07	0.79–1.45	0.656	1.13	0.46–2.77	0.784
Outside Europe (Reference)	1			1			1		
Occupational status			0.005 **			0.000 ***			0.056
Employed	2.63	1.46–4.76	0.001 ***	1.70	1.29–2.24	0.000 ***	1.39	0.73–2.65	0.318
Own business	1.79	0.78–4.08	0.170	2.25	1.67–3.04	0.000 ***	0.78	0.33–1.85	0.570
Student/Trainee	1.93	1.06–3.53	0.033 *	0.97	0.64–1.46	0.877	1.89	0.92–3.90	0.085
Leave of absence/Parental leave	2.54	1.15–5.63	0.021 *	1.07	0.74–1.56	0.716	2.04	0.86–4.85	0.107
Outside labor market	2.22	0.97–5.12	0.060	2.21	1.66–2.93	0.000 ***	0.95	0.40–2.28	0.908
Unemployed (Reference)	1			1			1		
Financial strain			0.007 **			0.000 ***			0.003 **
No financial strain	1.56	1.07–2.28	0.021 *	2.79	2.25–3.47	0.000 ***	0.53	0.35–0.80	0.002 **
Some financial strain	1.16	0.79–1.71	0.448	1.66	1.32–2.10	0.000 ***	0.67	0.43–1.04	0.072
Major financial strain (Reference)	1			1			1		
Housing			0.983			0.429			0.443
Own	0.98	0.74–1.30	0.877	1.20	0.90–1.60	0.207	0.78	0.53–1.15	0.213
Rented	0.97	0.73–1.30	0.858	1.18	0.88–1.58	0.273	0.83	0.56–1.25	0.377
Lodger/Dormitory/Other (Reference)	1			1			1		
Emotional support			0.000 ***			0.000 ***			0.072
High emotional support	1.95	1.08–3.53	0.027 **	1.91	1.62–2.27	0.000 ***	0.93	0.55–1.57	0.779
Moderate emotional support	1.14	0.63–2.05	0.672	1.49	1.27–1.74	0.000 ***	0.70	0.40–1.22	0.204
Low emotional support (Reference)	1			1			1		
Instrumental support			0.373			0.001 **			0.206
High instrumental support	1.03	0.41–2.58	0.954	1.45	1.19–1.78	0.000 ***	0.66	0.29–1.52	0.329
Moderate instrumental support	0.84	0.34–2.09	0.711	1.29	1.06–1.57	0.009 *	0.55	0.23–1.29	0.167
Low instrumental support (Reference)	1			1			1		
Interpersonal trust			0.001 **			0.033 *			0.018 *
Strong interpersonal trust	1.33	0.96–1.83	0.084	1.23	1.04–1.44	0.014 *	1.02	0.73–1.43	0.909
Fair interpersonal trust	0.87	0.65–1.16	0.345	1.14	0.98–1.33	0.089	0.75	0.55–1.03	0.072
Weak interpersonal trust (Reference)	1			1			1		
Community trust			0.864			0.044 *			0.699
Strong community trust	1.01	0.81–1.27	0.927	1.11	1.02–1.21	0.019 *	0.91	0.72–1.15	0.432
Fair community trust	1.07	0.83–1.38	0.598	1.08	0.98–1.19	0.112	1.00	0.76–1.31	0.996
Weak community trust (Reference)	1			1			1		
Societal participation									
Yes (Reference: did not participate)	0.94	0.75–1.19	0.611	0.97	0.90–1.05	0.496	0.93	0.74–1.16	0.505
Voting									
Yes (Reference: did not vote)	0.86	0.61–1.21	0.384	0.98	0.84–1.16	0.847	0.95	0.66–1.37	0.773
Nutrition—consumption of breakfast			0.548			0.829			0.994
Daily consumption of breakfast	1.26	0.81–1.96	0.303	1.06	0.87–1.31	0.561	1.03	0.64–1.64	0.912
Weekly consumption of breakfast	1.17	0.73–1.87	0.513	1.04	0.83–1.32	0.717	1.03	0.61–1.72	0.921
Seldom consumption of breakfast (Reference)	1			1			1		
Nutrition—consumption of fruit and berries			0.071			0.168			0.900
Daily consumption of fruit and berries	1.39	1.05–1.84	0.021 *	1.13	1.00–1.28	0.060	1.07	0.81–1.42	0.649
Weekly consumption of fruit and berries	1.25	0.94–1.67	0.127	1.09	0.95–1.25	0.207	1.04	0.77–1.42	0.784
Rare consumption of fruit and berries (Reference)	1			1			1		
Physical activity			0.438			0.000 ***			0.030 *
Regular physical activity	0.99	0.68–1.44	0.960	1.54	1.31–1.80	0.000 ***	0.65	0.44–0.94	0.023 *
Moderate physical activity	0.87	0.63–1.20	0.395	1.34	1.19–1.51	0.000 ***	0.65	0.47–0.90	0.010 *
Sedentary physical behavior (Reference)	1			1			1		
Tobacco smoking									
No (Reference: Daily smoking)	0.72	0.51–1.01	0.058	0.95	0.85–1.07	0.396	0.70	0.50–0.97	0.033 *
Hazardous alcohol consumption									
No (Reference: Hazardous alcohol consumption)	0.93	0.75–1.16	0.522	1.11	1.02–1.21	0.012 *	0.846	0.68–1.06	0.138
Victim of threat and violence			0.447			0.000 ***			0.736
Not victim of threat and violence	1.09	0.50–2.39	0.823	1.25	0.82–1.90	0.306	0.80	0.33–1.92	0.621
Victim of threat	0.80	0.33–1.92	0.611	0.74	0.46–1.21	0.229	1.04	0.39–2.83	0.934
Victim of violence	0.86	0.34–2.20	0.754	1.11	0.64–1.92	0.709	0.79	0.27–2.32	0.673
Victim of threat and violence (Reference)	1			1			1		

Data are presented as: OR, Odds Ratio; CI, Confidence Interval; reference value = 1. *p*-values indicate the following: *p* > 0.05 nonsignificant, * *p* ≤ 0.05, ** *p* ≤ 0.01, *** *p* ≤ 0.001.

## Supplementary Materials

The following are available online http://www.mdpi.com/1660-4601/15/12/2859/s1. Figure S1. Flow-chart of the 30 years and older age group in the Stockholm Public Health Cohort 2002, 2007, 2010, and 2014. Table S1. Strobe Statement—Checklist for Cohort Studies. Table S2. Potential determinants of health, survey items, response alternatives, categorization, and comments.
