# Supplementary material for: What Predicts Stable Mental Health in the 18–29 Age Group Compared to Older Age Groups? Results from the Stockholm Public Health Cohort 2002–2014
Source: Int J Environ Res Public Health. 2018 Dec 14;15(12):2859. doi: 10.3390/ijerph15122859 (PMC6313492; doi:10.3390/ijerph15122859)
Supplement: Supplementary file 1 [file ijerph-15-02859-s001.zip › Supplementary Materials_Table S2_Survey items.pdf]

Supplementary Materials, Table S2. Potential determinants of health, survey items, response alternatives, categorization, and comments.

Presumptive determinants of health	Survey items	Response alternatives	Categorization	Comments
**Sociodemographic factors**				
**Gender**	----	----	1 Male<br>2 Female [Reference]	Register data. Obtained from Statistics Sweden (http://www.scb.se).
**Country of birth**	----	----	1 Sweden<br>2 Other Nordic countries<br>3 Other Europe<br>4 Outside Europe [Reference]	Register data. Obtained by linkage with the Longitudinal Integration Database for Health Insurance and Labour Market Studies (LISA), held by Statistics Sweden (http://www.scb.se).
**Housing**	In what type of housing do you live?	1 Own house or terrace house<br>2 Tenant-owned (flat or terrace house)<br>3 Rental<br>4 Lodger/dormitory<br>5 Other type of housing	1 Own = Own house/terrace house/Tenant-owned<br>2 Rental<br>3 Other = Lodger/dormitory + Other type of housing [Reference]	
**Occupational status**	Which of the following alternatives apply to you right now?	1 Employment<br>2 Own business<br>3 Disability pensioner<br>4 Old age or contractual pensioner<br>5 Leave of absence or<br>parental leave,<br>6 Student/trainee<br>7 Unemployed<br>8 Other	1 Employment<br>2 Own business<br>3 Student/trainee<br>4 Leave of absence or parental leave<br>5 Outside labour-market =Disabilty pensioner + Old age or contractual pensioner + Other<br>6 Unemployed [Reference]	
**Economic strain**	A Has it happened during the past 12 months that you had to borrow money from relatives or friends to manage current	1 No<br>2 Yes, once<br>3 Yes, several times	1 No financial strain<br>2 Some financial strain<br>3 Major financial strain [Reference]	A merging of item A and B

	expenditures for food and rent?			
	B Have you, in the past 12 months refrained from going to the dentist, medical services or picking up prescriptions as a result of poor finances? Several alternatives may be indicated.	1 No<br>2 Yes, the dentist due to poor finances<br>3 Yes, medical services due to poor finances<br>4 Yes, picking up prescriptions due to poor finances	1 No economic strain<br>2 Some economic strain (1 Yes- alternative)<br>3 Major economic strain (2 or 3 Yes-alternative) [Reference]	
**Support, Trust, Participation**				
**Emotional support**	Do you know any people who can provide you with personal support for personal problems or crises in your life?	1 Yes, always<br>2 Yes, most of the time<br>3 No, mostly not<br>4 No, never	1 High =Yes, always<br>2 Moderate = Yes, most of the time<br>3 Low = No, mostly not + No, never [Reference]	
**Instrumental support**	Can you get help from another person/other persons if you have practical problems or are ill?	1 Yes, always<br>2 Yes, most of the time<br>3 No, mostly not<br>4 No, never	1 High =Yes, always<br>2 Moderate = Yes, most of the time<br>3 Low = No, mostly not + No, never [Reference]	
**Interpersonal trust**	How well do you think the following statement describes the place where you live? You can trust most of the people living in your neighbourhood?	1 Very accurate<br>2 Fairly accurate<br>3 Not particularly accurate<br>4 Not at all	1 Strong = Very accurate<br>2 Fair = Fairly accurate<br>3 Weak = Not particularly accurate + Not at all [Reference]	
**Community trust**	How much confidence do you have in the following public institutions?	1 Considerable<br>2 Fairly considerable<br>3 Little<br>4 None whatsoever	Strong = Considerable<br>Fair = Fairly consideralble<br>Weak = Little +None whatsosever [Reference]	Summary variable categorized. Maximum points: 36.<br>1 – 24 Strong<br>25 – 27 Fair

	a) Medical services<br>b) Social services<br>c) The National insurance office<br>d) The Official employment services<br>e) The Police<br>f) The Parliament<br>g) The Government<br>h) Politicians in your<br>region<br>i) Polititicians in yor municipality			28 – 36 Weak [Reference]
**Societal participation**	In the past 12 months, have you more or less regularly participated in activities together with several other people? For example sport, music/theatre, courses, religious gatherings, choir, sewing groups, political associations or other society.	1 Yes<br>2 No	1 Yes<br>2 No [Reference]	
**Voting**	Did you vote in any election in 2002? That is parliamentary, county health authority or municipal elections.	1Yes<br>2 No	1Yes<br>2 No [Reference]	
**Healthy diet, Physical activity**				
**Nutrition – consumption of breakfast**	How often do you eat breakfast?	1 = Daily or almost daily<br>2= 3-5 times per week<br>3= Once – twice per week<br>4= Seldom or never	1 Daily = Daily or almost daily<br>2 Several times a week = 3-5 times per week + Once – twice per week<br>3 Seldom or never [Reference]	Consumption of breakfast and fruit and Berries should be seen as proxies for healthy diet due to the

				unavailability of other diet items 2002.
**Nutrition – consumption of fruit and berries**	How often do you eat Fruit and berries (an apple, an orange, a banana, a glass of juice, grapes, strawberries)	1= Several times a day<br>2 =Daily<br>3= A few times a week<br>4= Once a week<br>5=1-3 times per month<br>6= Never or nearly never	1 Daily = Several times a day + Daily<br>2 Several times a week =A few times a week + Once a week<br>3 Seldom = 1-3 times per month + Never or nearly never [Reference]	
**Physical activity**	State your physical exercise in your leisure time during the last 12 months. The level may vary over the course of the year or a week, but try to give an average.	1 Regular = On average 3 times per week at least 30 minutes<br>2 Moderate, regularly = Once-twice per week, at 30 minutes, sweating<br>3 Moderate = At least 2 hours per week, usually without sweating<br>4 Sedentary leisure time = Less than 2 hours per week	1 Regular<br>2 Moderate = Moderate regularly + Moderate<br>3 Sedentary [Reference]	
**Alcohol, Gambling, Smoking**				
**Hazardous alcohol consumption**	What are your alcohol habits during a typical week? Try to state an average.	A table including week-days Monday – Sunday and the number of drinks for spirit, fortified wine, wine, strong cider/alcopop, strong beer, and medium strong beer is to be filled in.	Gram pure alcohol per week, computed by Statistics Sweden.<br>>108 g per week for women (approx. 9 standard glasses)<br>> 168 g per week for men<br>(approx. 14 standard glasses).<br>1 = no risk <108 g and < 168 g, respectively<br>2 = risk >108 g and > 168 g, respectively [Reference]	
**Tobacco smoking**	Do you smoke daily i.e. cigarettes, pipe, cigars and/or cigarillos?	1 No<br>2 Yes	1 No<br>2 Yes [Reference]	
**Violations**				
**Physical violence**	Have you during the past 12 months been a victim of	1 No<br>2 Yes	1= Not subject to a threat of violence or physical violence	

	physical violence?		2= Subject to threat of violence<br>3= Victim of physical violence<br>4= Subject to threat of violence and victim of physical violence. [Reference]
**Threat of violence**	Have you at any point in time in the past 12 months been subject to a threat of violence so intimidating or serious that you were frightened?	1 No<br>2 Yes	
