# Supplementary material for: What Predicts Stable Mental Health in the 18–29 Age Group Compared to Older Age Groups? Results from the Stockholm Public Health Cohort 2002–2014
Source: Int J Environ Res Public Health. 2018 Dec 14;15(12):2859. doi: 10.3390/ijerph15122859 (PMC6313492; doi:10.3390/ijerph15122859)
Supplement: Supplementary file 1 [file ijerph-15-02859-s001.zip › Supplementary Materials_ Figure S1_ Flow chart_ 30 years_older.docx]

Attrition n=251

Attrition n=3332

Attrition n=4015

**Supplementary Materials, Figure S1.** Flow-chart of the respondents in the age group 30 years and older of the Stockholm Public Health Cohort 2002, 2007, 2010, and 2014.
